# Fusion of High-Dynamic and Low-Drift Sensors Using Kalman Filters

**DOI:** 10.3390/s19010186

**Published:** 2019-01-07

**Authors:** Bin Wu, Tiantian Huang, Yan Jin, Jie Pan, Kaichen Song

**Affiliations:** 1 College of Biomedical Engineering & Instrument Science, Zhejiang University, Hangzhou 310027, China; wubinzju@zju.edu.cn (B.W.); jinyanchn@zju.edu.cn (Y.J.); kcsong@zju.edu.cn (K.S.); 2 Department of Mechanical Engineering, The University of Western Australia, 35 Stirling Highway, Crawley, WA 6009, Australia

**Keywords:** dual sensor system, high dynamic, low drift, Kalman filter

## Abstract

In practice, a high-dynamic vibration sensor is often plagued by the problem of drift, which is caused by thermal effects. Conversely, low-drift sensors typically have a limited sample rate range. This paper presents a system combining different types of sensors to address general drift problems that occur in measurements for high-dynamic vibration signals. In this paper, the hardware structure and algorithms for fusing high-dynamic and low-drift sensors are described. The algorithms include a drift state estimation and a Kalman filter based on a linear prediction model. Key issues such as the dimension of the drift state vector, the order of the linear prediction model, are analyzed in the design of algorithm. The performance of the algorithm is illustrated by a simulation example and experiments. The simulation and experimental results show that the drift can be removed while the high-dynamic measuring ability is retained. A high-dynamic vibration measuring system with the frequency range starting from 0 Hz is achieved. Meanwhile, measurement noise was improved 9.3 dB through using the linear-prediction-based Kalman filter.

## 1. Introduction

For mining machines, measuring vibrations is an important way to detect early failure features [1]. It requires the measuring system to provide a high sample rate in order to analyze the high-order modes up to 1.75 kHz [2]. However, the measuring system also needs to have a low-drift characteristic in order to detect the low-frequency signal, since the mining machines usually work at extremely low rotation speeds [3]. These two requirements are difficult to meet simultaneously for a single type of sensor. Piezoelectric sensors are widely used in vibration monitoring systems, thanks to their advantages of high resolution, low background noise, and wide frequency band [4]. However, their disadvantage is bias drift caused by thermal effects [5]. Feedback-based sensors, such as the quartz flexure accelerometer, can measure static acceleration because of their low thermal drift [6], but their frequency band is narrower than that of a piezoelectric sensor. Hence, to meet the requirements of combined high-dynamic and low-drift vibration measurement, this paper proposes a dual-sensor-based hardware structure and the associated processing algorithms.

Using multiple sensors to extract a broadband vibration signal has been studied by many researchers and shown to be feasible [7,8,9]. Traditionally, sensors of the same type are used, which leads to nearly identical noise and drift characteristics [10]. The improvement in noise and drift is proportional to the square root of the number of sensors. That means the number of sensors used in such a system would grow rapidly as the requirement for improvement becomes higher. To overcome this drawback, the current trend is to fuse different types of sensors. For example, Safizadeh and Latifi propose a method fusing an accelerometer and a load cell [11]. Park et al. develop a system incorporating time synchronized acceleration and strain measurements [12]. These work fuse sensors measuring different physical quantities. This paper focuses on a vibration-measuring system incorporating two different types of accelerometers that have different noise characteristics. One sensor works at a high sample rate to provide the dynamic performance but its output is more sensitive to thermal effects, while the other sensor works at a low sample rate to achieve low-drift quality but it has narrower frequency range. As these two different sensors are combined, the frequency range of the vibration measuring system extends from the ultra-low frequency to the maximum frequency of the high-dynamic sensor. This combined use of different sensors requires a suitable algorithm to fuse the measurement data.

There are various fusion algorithms used in the multi-sensor measuring field [13]. Among them, the complementary filter is widely used because it requires less computation. However, it is designed using a simple analysis in the frequency domain and it does not consider any statistical property of the noise [14]. The Kalman filter, which is commonly adopted in navigation systems [15,16,17,18], has received much attention from the multi-sensor measuring field during the last 50 years [19]. For example, a system based on two odometers and a magnetic compass is developed to determine the position and orientation of a wheelchair in indoor environments, and a comparative study of measurement fusion (MF) and state vector fusion (SVF) is proposed [20]. In the MF structure, measurements from different sensor are combined before applying the Kalman filter. While in the SVF structure, measurements from different sensors are processed separately and the resulting state vectors are merged to obtain a final state vector. Analysis shows that the MF structure requires less computation than the SVF structure [21]. Considering the realization in embedded systems, this paper chooses the MF structure to fuse measurements from the high-dynamic sensor and the low-drift sensor in the first stage, where the drift of the high-dynamic sensor is calibrated. In the second stage, the drift-removed data are processed by a linear-prediction-based Kalman filter for improving measurement noise. The linear prediction model assumes that the current data can be predicted by several previous data in time series. As a comparison, the well-known random walk model assumes that the current data is equivalent to the last data [22]. This paper found that the prediction error of the linear prediction model was smaller than the random walk model, hence the sample rate of linear-prediction-based Kalman filter could be lower than the random-walk-based Kalman filter.

The rest of this paper is organized as follows: Section 2 describes the block diagram of the dual-sensor vibration measuring system and presents the details of the processing algorithms. Section 3 analyzes the key factors for algorithm design. The performances of the proposed hardware structure and algorithms are investigated through simulations in Section 4. Experiments and related discussions are presented in Section 5. Finally, Section 6 provides the conclusions.

## 2. Dual-Sensor Vibration Measuring System

**Figure 1:**
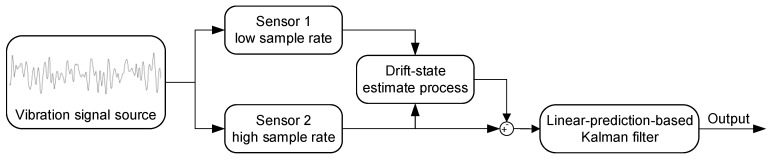
Block diagram of the hardware structure and the data-fusion algorithm.

The frame of hardware structure and the processing algorithms is shown in Figure 1. Two types of sensors are attached onto the vibration signal source (mining machines) to measure the same acceleration. One is a high-dynamic sensor (piezoelectric sensor) that works at a high sample rate, the other is a low-drift sensor (quartz flexure accelerometer) that works at a low sample rate. 

The sample rates of two sensors are synchronized and fixed at a constant ratio. The sample rate of the low drift sensor can be lower than the Nyquist frequency of the vibration signals. This is because the low-drift sensor is used to calibrate the drift in the high-dynamic sensor. Typically, drift changes slowly over a long period. Consequently, the sample rate of a low-drift sensor can be very low.

**Figure 2:**
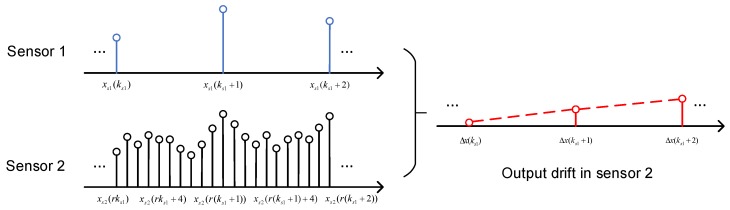
Illustration of the drift-state estimation process.

The algorithms include a drift-state estimation process and a linear-prediction-based Kalman filter. The drift-state estimation process utilizes the low-sample-rate data and the corresponding high-sample-rate data to obtain the drift model caused by thermal effects in the high-dynamic sensor, and then calibrate the drift of that high-dynamic sensor. The details are shown in Figure 2. In this process, the low-sample-rate data (Sensor 1) is expressed as:

(1) $$x_{s1}\left(k_{s1}\right)=s_{s1}\left(k_{s1}\right)+n_{s1}\left(k_{s1}\right)$$

and the corresponding high-sample-rate data (Sensor 2) is expressed as:

(2) $$x_{s2}\left(rk_{s1}\right)=s_{s2}\left(rk_{s1}\right)+n_{s2}\left(rk_{s1}\right)+d_{s2}\left(rk_{s1}\right),$$

where $k_{s1}$ is the index of the data obtained from the low-sample-rate sensor; r is the ratio of the high sample rate to the low sample rate; $s_{s1}$ and $s_{s2}$ are the vibration signals measured by the low-sample-rate sensor and the high-sample-rate sensor, respectively; $n_{s1}$ and $n_{s2}$ are the measurement noise (band-limited white noise) of the low-sample-rate sensor and the high-sample-rate sensor, respectively; and $d_{s2}$ is the output drift of Sensor 2. Subtracting Equation (1) from Equation (2), the difference between data from Sensor 1 and Sensor 2 is given by:

(3) $$\Delta x\left(k_{s1}\right)=d_{s2}\left(rk_{s1}\right)+\left(n_{s2}\left(rk_{s1}\right)-n_{s1}\left(k_{s1}\right)\right),$$

as the signal component $s_{s1}\left(k_{s1}\right)$ measured by Sensor 1 is equivalent to that measured by Sensor 2, $s_{s2}\left(rk_{s1}\right)$. In Equation (3), the differential data $\Delta x\left(k_{s1}\right)$ is hold at the sample time of the Sensor 1.

The aim of this drift-state estimation process is to give the best estimation of the drift in the high-sample-rate sensor $d_{s2}\left(k_{s2}\right)$ (where $k_{s2}$ denotes the index of the data obtained from the high-sample-rate sensor) based on the differential data $\Delta x\left(k_{s1}\right)$, which is a combination of the down-sampled drift data $d_{s2}\left(rk_{s1}\right)$ and the measurement noise ($n_{s2}\left(rk_{s1}\right)-n_{s1}\left(k_{s1}\right)$). It is assumed that the drift data can be fitted by a polynomial function. In time domain, the drift of high-dynamic sensor can be expressed as:

(4) $$d_{s2}\left(t\right)=C_{q}t^{q}+C_{q-1}t^{q-1}+\cdots +C_{1}t..\;$$

The coefficients of that polynomial function are treated as the state vector. To estimate this drift state, a Kalman filter based on random-walk model is applied. In this Kalman filter, the system state vector is composed of polynomial function coefficients, which are arranged in descending order, as shown below:

(5) $$C={\left[\begin{array}{cccc} c_{q} & c_{q-1} & \ldots \; & c_{1} \end{array}\right]}^{T},$$

where $c_{q}$ is the coefficient of the qth-order term. The constant coefficient $c_{0}$ is not considered here since the measuring system starts from a zeroed initial state. Choosing the dimension of the state vector is a trade-off between prediction error and computation time.

The observation matrix is given by:

(6) $$H_{k_{s1}}=\left[\begin{array}{cccc} {\left(\frac{k_{s1}}{f_{s1}}\right)}^{q} & {\left(\frac{k_{s1}}{f_{s1}}\right)}^{q-1} & \ldots & \left(\frac{k_{s1}}{f_{s1}}\right) \end{array}\right],$$

where $f_{s1}$ denotes the low sampling frequency and $k_{s1}$ denotes the index of the low-sample-rate data. Hence, the result $\frac{k_{s1}}{f_{s1}}$ represents the discrete time $t_{k_{s1}}$. The observation value $\Delta x\left(k_{s1}\right)$ is given in Equation (3). Equation (3) shows that the covariance matrix $R_{k_{s1}}$ is affected by low-sample-rate measurement noise as well as high-sample-rate measurement noise. Finally, the calibrated state estimate can be expressed as:

(7) $${\hat{C}}_{k_{s1}|k_{s1}}={\hat{C}}_{k_{s1}|k_{s1}-1}+K_{k_{s1}}\left(\Delta x\left(k_{s1}\right)-H_{k_{s1}}{\hat{C}}_{k_{s1}|k_{s1}-1}\right),$$

where ${\hat{C}}_{k_{s1}|k_{s1}-1}$ is the predicted state, and $K_{k_{s1}}$ is the optimal Kalman gain.When the low-drift sensor measures a new datum, the state estimate and the covariance matrix of the estimate error will be updated. Once the new estimate is generated, the drift for the following (r−1) points of high-sample-rate data can be calculated as:

(8) $${\hat{d}}_{s2}\left(k_{s2}\right)=H_{k_{s2}}{\hat{C}}_{k_{s1}|k_{s1}},$$

where the observation matrix $H_{k_{s2}}$ can be expressed as:

(9) $$H_{k_{s2}}=\left[\begin{array}{cccc} {\left(\frac{k_{s2}}{f_{s2}}\right)}^{q} & {\left(\frac{k_{s2}}{f_{s2}}\right)}^{q-1} & \;\ldots & \left(\frac{k_{s2}}{f_{s2}}\right) \end{array}\right],$$

where $k_{s2}$ is the index of the high-sample-rate data and $f_{s2}$ denotes the sample frequency of the high-sample-rate sensor. Hence, the result $\frac{k_{s2}}{f_{s2}}$ represents the discrete time $t_{k_{s2}}$. Subtracting ${\hat{d}}_{s2}\left(k_{s2}\right)$ from $x_{s2}\left(k_{s2}\right)$ removes the drift part of the high-sample-rate data:

(10) $${\hat{x}}_{s2}\left(k_{s2}\right)=x_{s2}\left(k_{s2}\right)-{\hat{d}}_{s2}\left(k_{s2}\right).$$

**Figure 3:**
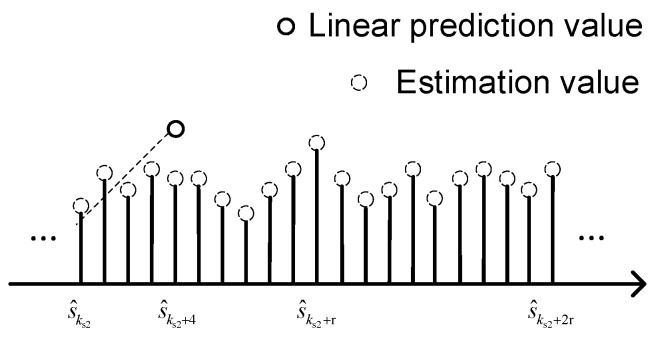
Illustration of the linear prediction model.

After the drift is removed, a linear-prediction-based Kalman filter is proposed to reduce measurement noise. This linear-prediction-based Kalman filter works on the time series obtained from the previous process, it is developed from a random-walk forecast model. The random-walk forecast model assumes that the current state is equivalent to the last state, where state means true value of the current data. This assumption is reasonable when the data are sampled at a rate that is much faster than the dynamic of the state variable. However, this assumption is invalid when the sample rate is not sufficiently high relative to the dynamic signal. For this case the linear-prediction-based Kalman filter is more appropriate than the random-walk forecast model. The linear-prediction-based Kalman filter, as shown in Figure 3, assumes that the current data can be predicted by a linear fitting of several previous data. For example, for a fourth-order linear prediction model, the fifth signal datum is predicted from four previous signal data. Since the real signal is unknown, the estimate is used instead. Each estimate is generated by the corresponding Kalman filter iteration.

As the core of this Kalman filter, the state variable $S_{k_{s2}}$ of the linear-prediction-based Kalman filter is given by:

(11) $$S_{k_{s2}}={\left[s_{k_{s2}}\;s_{k_{s2}-1}\;\cdots s_{k_{s2}-p}\right]}^{T},$$

where $s_{k_{s2}}$ is the $k_{s2}$th signal data and p is the order of the linear fitting equation. The state transmission equation can be expressed as:

(12) $$\left[\begin{array}{c} s_{k_{s2}}\\ s_{k_{s2}-1}\\ \vdots \\ s_{k_{s2}-p} \end{array}\right]=\left[\begin{array}{cccc} c_{1} & c_{2} & \cdots & c_{p}\\ 1 & 0 & \cdots & 0\\ 0 & 1 & \cdots & 0\\ 0 & 0 & \cdots & 0 \end{array}\right]\left[\begin{array}{c} s_{k_{s2}-1}\\ s_{k_{s2}-2}\\ \vdots \\ s_{k_{s2}-\left(p+1\right)} \end{array}\right],$$

where $c_{j}$ is the linear-fitting coefficient. For a *p*-th order linear prediction model, the current signal datum $s_{k_{s2}}$ is a combination of the p previous signal data and the *j*-th combination coefficient:

(13) $$c_{j}=\left(\frac{6}{-p^{2}+p}-\frac{12\left(p+1\right)}{-p^{3}+p}\right)\left(p+1-j\right)+\frac{2p+4}{-p^{2}+p},\;j\geq 2.$$

In the updating process for the linear-prediction-based Kalman filter, the state transmission equation can also be expressed in a matrix form:

(14) $${\hat{S}}_{m|m-1}=F_{m}{\hat{S}}_{m-1|m-1},$$

where ${\hat{S}}_{m|m-1}$ denotes the predicted state estimate, $F_{m}$ is the state transmission matrix shown in Equation (15), and ${\hat{S}}_{m-1|m-1}$ denotes the best estimate given by the previous step. Hence, the covariance matrix $\Sigma_{S_{k_{s2}|k_{s2}-1}}$ can be obtained by:

(15) $$\Sigma_{k_{s2}|k_{s2}-1}=F_{k_{s2}}\Sigma_{k_{s2}-1|k_{s2}-1}F_{k_{s2}}^{T}+Q_{k_{s2}},$$

where $\Sigma_{k_{s2}|k_{s2}-1}$ and $\Sigma_{k_{s2}-1|k_{s2}-1}$ are the covariance matrix of the prediction error and the last Kalman estimation, respectively. Based on the covariance matrix $\Sigma_{k_{s2}|k_{s2}-1}$, the gain factor is given by:

(16) $$K_{k_{s2}}=\Sigma_{k_{s2}|k_{s2}-1}A_{k_{s2}}^{T}{\left(R_{k_{s2}}+A_{k_{s2}}\Sigma_{k_{s2}|k_{s2}-1}A_{k_{s2}}^{T}\right)}^{-1},$$

where $A_{k_{s2}}$ is the measurement matrix and $R_{k_{s2}}$ is the covariance matrix of the measurement error. The measurement matrix converts the state variable into an observable vector. In this paper, the state variable is measured directly, hence the measurement matrix is an identity matrix. The estimation given by the Kalman filter can be expressed as:

(17) $${\hat{S}}_{k_{s2}|k_{s2}}={\hat{S}}_{k_{s2}|k_{s2}-1}+K_{k_{s2}}\left(b_{k_{s2}}-A_{k_{s2}}{\hat{S}}_{k_{s2}|k_{s2}-1}\right),$$

where $b_{k_{s2}}$ is the measurement vector. Each measurement vector is extracted from ${\hat{x}}_{2}$ and has the same dimension as the state variable $S_{k_{s2}}$. The difference between the measurement and prediction values is amplified by the Kalman gain, which is used to correct the prediction value and then generate the new estimation. The covariance matrix of the new estimation is given by:

(18) $$\Sigma_{k_{s2}|k_{s2}}=\left(I-K_{k_{s2}}A_{k_{s2}}\right)\Sigma_{k_{s2}|k_{s2}-1}.$$

## 3. Analysis of Key Factors

For the drift-state estimation process, the sample frequency of the low-sample-rate sensor depends on the drift rather than the vibration signal. According to practical experience [23,24], sensor drift is a long-term variable that changes very slowly, and hence the requirement for the sample frequency of the low-sample-rate sensor is easily satisfied according to the Nyquist–Shannon sampling theorem. There is a possibility that the sample frequency of the low-sample-rate sensor is lower than the highest signal frequency, which will lead to an aliasing problem in the low-sample-rate data. This problem is solved by subtracting low-sample-rate data from high-sample-rate data, which leaves only the drift part to estimate. Consequently, no anti-aliasing filter is required before the low-sample-rate sensor. Since the required sample frequency for the low-sample-rate sensor can be very low, some non-drift sensors, which are unable to achieve high sample frequencies, can be used for this application.

The dimension of the state vector decides the order of the polynomial function used to fit the drift of the high dynamic sensor. The drift phenomenon depends on various stochastic factors. An alternative model to fit the thermal drift is an exponential function [25]. However, this exponential function needs to be linearized with Taylor-series expansions when used in an extended Kalman filter, which increases the computation load. Here, a 5th-order polynomial model to fit the thermal drift is investigated. The exponential model can be expressed as:

(19) $$d\left(t\right)=C_{1}\left(1-e^{-\frac{t}{T}}\right)+C_{2},$$

where $C_{1}$, $C_{2}$, and T are parameters to be tuned. The Taylor-series expansion of Equation (22) at t=0 can be written as:

(20) $$d\left(t\right)=C_{2}+\frac{C_{1}}{T}t-\frac{C_{1}}{2T^{2}}t^{2}+\frac{C_{1}}{6T^{3}}t^{3}-\frac{C_{1}}{24T^{4}}t^{4}+R_{4}\left(t\right),$$

where $R_{4}\left(t\right)$ is the residual function. For $t\in \left[0,t_{0}\right]$, $R_{4}\left(t\right)$ is limited by:

(21) $$R_{4}\left(t\right)\leq \frac{M_{n}{\left(\frac{t_{0}}{2}\right)}^{5}}{5!},$$

where $M_{n}$ is a number such that:

(22) $$\left|\frac{C_{1}}{120T^{5}}e^{-\frac{t}{T}}\right|\leq M_{n}$$

for any $t\in \left[0,t_{0}\right]$. Hence, for a short time period from time 0 to time $t_{0}$, the residual can be controlled in a limited range. This period depends on how long the drift state remains stable, and the order of the polynomial function can be adjusted according to the period.

The current state vector of drift is assumed to be equivalent to the previous one as a random-walk model is applied in the state transmission equation. The covariance matrix of the state transmission error depends on the consistency of the drift characteristic during operation [26]. This covariance matrix is closely related to the specific vibration sensors and the operation environment.

The covariance matrix of observation error was demonstrated in Equation (3). Suppose the standard deviation of $n_{1}\left(k\right)$ is $\sigma_{1}$ and the standard deviation of $n_{2}\left(m\right)$ is $\sigma_{2}$, where $n_{1}\left(k\right)$ and $n_{2}\left(m\right)$ denote the measurement noises of the low-drift sensor and the high-dynamic sensor, respectively. The covariance matrix of observation error can be written as:

(23) $$R_{k}=\sigma_{1}^{2}+\sigma_{2}^{2}.$$

With all the above four factors considered, the optimal drift estimate is generated to calibrate the high-sample-rate data. Given the drift-removed data of the high-dynamic sensor, a linear-prediction-based Kalman filter is applied to reduce the measurement noise. Two key parameters in this Kalman filter are the covariance matrix of the prediction error and the covariance matrix of the measurement error.

**Figure 4:**
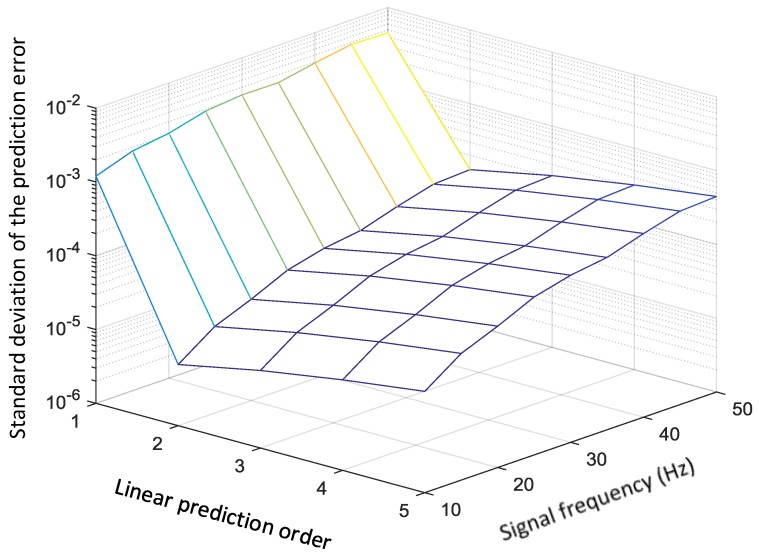
The effect of linear prediction order and signal frequency on prediction error.

Given the sample frequency of the high-dynamic sensor, the covariance matrix of the prediction error is influenced by the order of the linear prediction model and the signal frequency. For a pth-order linear prediction model, each current datum $s_{m}$ is a combination of p previous data and the *j-*th combination coefficient is as shown in Equation (13). The simplest case is p=1. In this case, to achieve a prediction error smaller than 0.001, the ratio of the sample rate to the signal frequency must be larger than 1000. The solution to this problem is to increase the order of the prediction model. A simulation result indicating the effect of the linear prediction order and signal frequency on prediction error is shown in Figure 4. In this figure, the sample rate is fixed at 10 kHz. Band-limited random signals, whose highest frequency changes from 10 Hz to 50 Hz, are used to compare the prediction errors of different orders.

The second-order linear prediction model greatly improved the prediction error compared to the random-walk model (p=1). However, for higher-order linear prediction models, the prediction error is increased rather than decreased. This can be explained by the transfer function of the linear prediction model. This model is equivalent to a low-pass filter [27]. The error is determined by the gain of the transfer function in the frequency band of interest. Investigation of the low-frequency band shows that the second-order model has smaller prediction errors than higher orders. Considering the additional computational load when the model order is increased, the optimal choice is to use a second-order linear prediction model.

When the band of the signal is broadened, the prediction errors of all models also increase if the sample rate remains the same. The reason is that the prediction value cannot follow the high-frequency component in the vibration signal. When the prediction error becomes as large as the measurement error, it will degrade the performance of the Kalman filter.

## 4. Simulation

**Figure 5:**
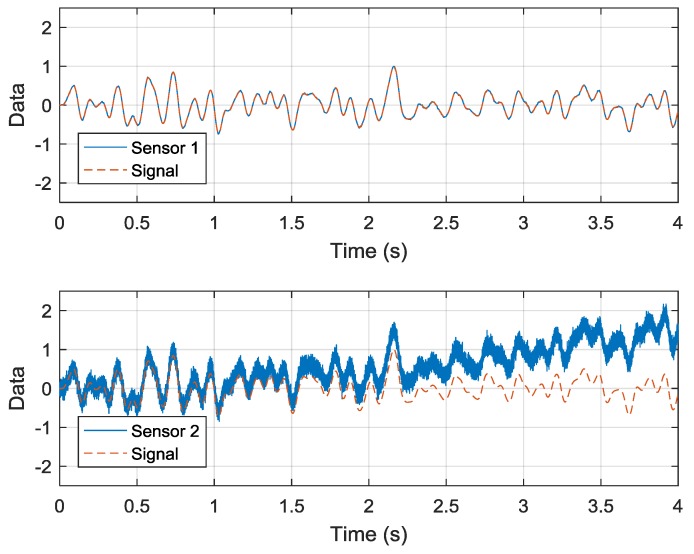
Simulation of the low-sample-rate data and high-sample-rate data.

To demonstrate the validity of the dual-sensor measuring system, a simulation is performed and the results are shown in Figure 5. In this simulation, Sensor 1 is the low-drift sensor and Sensor 2 is the high-dynamic sensor, whose sample rates are 200 Hz and 10 kHz, respectively. Corresponding to the different sample rates, the two kinds of sensors have different noise characteristics. The data of Sensor 1 are polluted by a band-limited white noise whose standard deviation is 0.01, while the data of Sensor 2 are polluted by a band-limited white noise and a thermal noise. The standard deviation of the white noise in Sensor 2 is 0.1. The thermal noise influences output drift.

The output drift of Sensor 2, denoted $d_{t}$, is designed to be a polynomial function of time, like so:

(24) $$d_{t}=1.2\times 10^{-1}\times t+3.1\times 10^{-2}\times t^{2}+6.5\times 10^{-3}\times t^{3}+8.7\times 10^{-4}\times t^{4}.$$

Both the white noise and the output drift are additive. For clarity, the frequency of the original signal is limited below 10 Hz. The amplitude of the original signal is normalized as 1. The data of Sensor 1 includes the original signal and the smaller white noise. The data of Sensor 2 is the sum of the original signal, the larger white noise, and the drift. The results for Sensor 2 show that the measurement data contain more noise and a time-varying drift. This time-varying drift in the high-sample-rate sensor is processed using the algorithm presented in Section 2.

**Figure 6:**
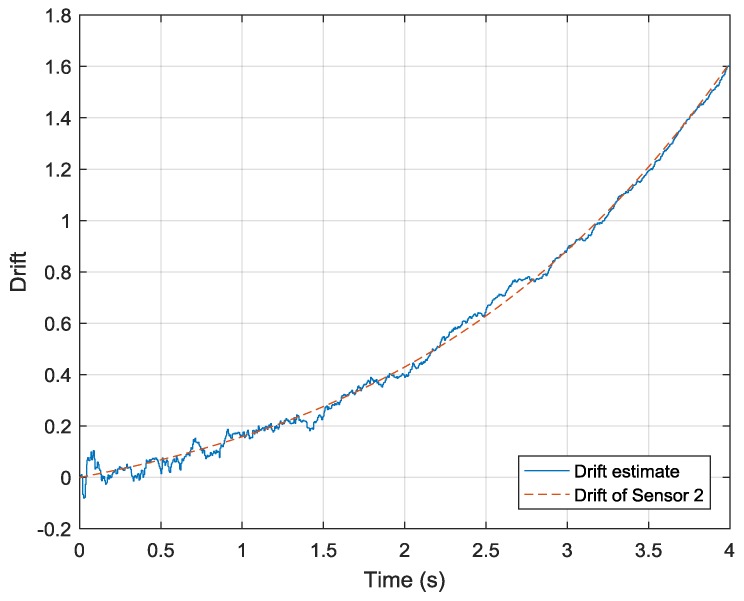
Drift-state estimation process.

The simulation result shown in Figure 6 indicates that this estimate converges to the true value given in Equation (24). In this simulation, the covariance matrix of the state transmission error is assigned very small values ($10^{-13}$ along the diagonals and 0 elsewhere) because the coefficients of Equation (24) are constant. The covariance matrix of observation error is assigned a single value of $1.01\times 10^{-2}$. With this estimate result, the drift-removed and high-dynamic data can be obtained.

**Figure 7:**
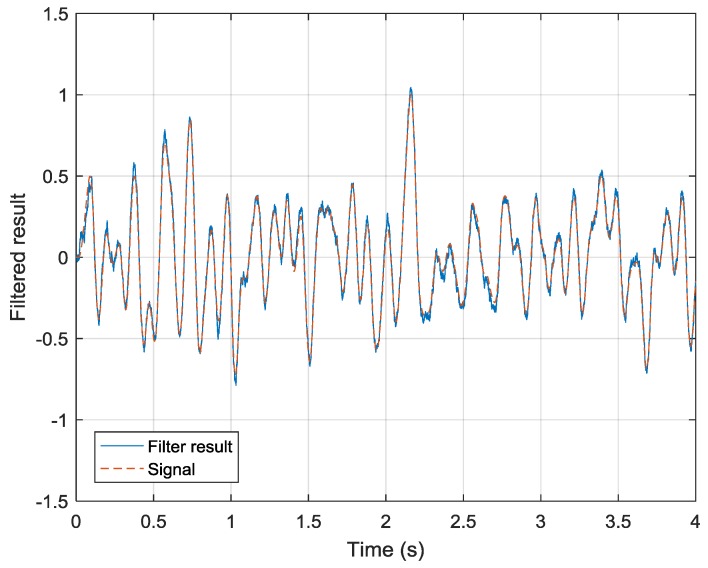
Simulation of the linear-prediction-based Kalman filter.

Following the drift-state estimation process, the drift-removed data of Sensor 2 are then processed using a linear-prediction-based Kalman filter. Two parameters are considered in this stage: the covariance matrix of the state transmission error and the covariance matrix of the observation error. The covariance matrix is adjusted according to the linear prediction order and the signal frequency band, which is shown in Figure 4. The observation error is decided by the measurement noise of Sensor 2, which is $10^{-2}$ in this simulation. The simulation result is shown in Figure 7. The simulation result shows that the output drift has been removed and the measurement noise has improved 9.3 dB (compared with that in Figure 5b). The root-mean-square error (RMSE) of the filtering result is 0.0342. In addition, the latency of the linear-prediction-based Kalman filter is 1 sample. This would be a useful characteristic when the system is used in a feedback control loop.

**Figure 8:**
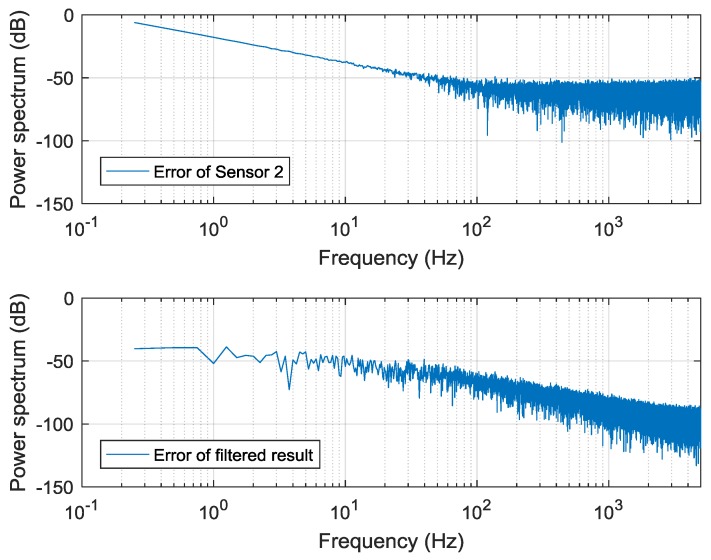
Power spectra of the errors.

Figure 8 shows the improvement of this algorithm. The first curve is the original error in Sensor 2. The second curve is the error of the filtered result. The simulation shows that a 34-dB improvement is achieved in the low-frequency band. The measurement noise in the high-frequency band is also reduced.

As the range of the signal band becomes wider, the anti-drift performance of the algorithms is not affected. The improvements for different signal bands are shown in Table 1.

## 5. Experiments and Discussion

**Figure 9:**
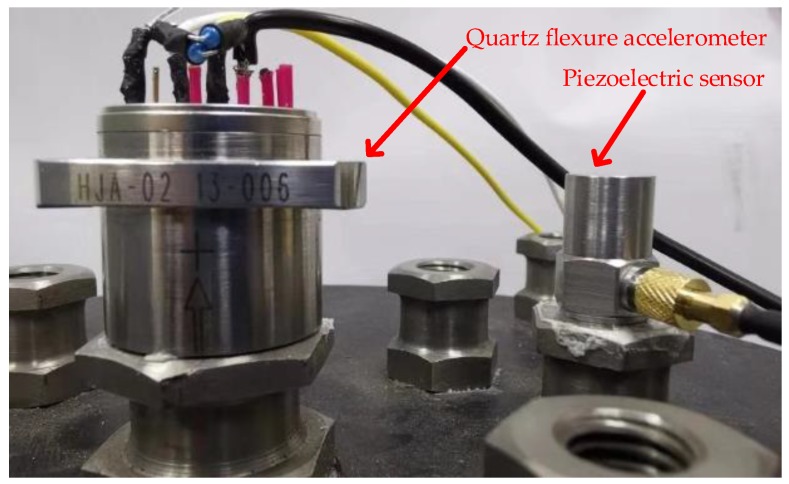
Experiment of the dual-sensor vibration measuring system.

To verify our method, a vibration measuring system involving two different kinds of sensors is shown in Figure 9. The high-dynamic sensor is a model CA-YD-103 piezoelectric sensor. The sensitivity of CA-YD-103 is 20 pC/g, and the frequency range is from 0.5 Hz to 12 kHz. Hence, in the application of ultra-low signal detection that required by mining machines, this common vibration sensor is unable to meet measuring requirements. This piezoelectric sensor is connected to a model DCA103 charge amplifier for obtaining voltage signals. The sensitivity of the DCA103 is 2.5 mV/pC. The low-drift sensor is a model HJA-02 quartz flexure accelerometer. The sensitivity of the HJA-02 is 2 mA/g, and the frequency range is from 0 Hz to 200 Hz. A 50 Ω resistance is used to sample the current output of HJA-02. The analog outputs of these two sensors are sampled by an oscilloscope (TBS 1102). These two sensors are fixed at a DC-1000-15 shaking table. In this fixing way, the input axis of piezoelectric sensor is opposite to the input axis of quartz flexure accelerometer.

**Figure 10:**
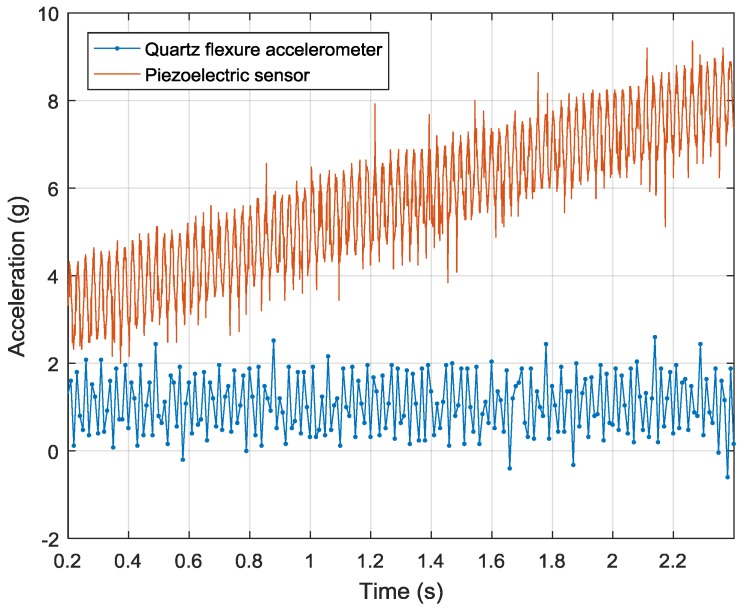
Original data of the quartz flexure accelerometer and piezoelectric sensor.

The original data of quartz flexure accelerometer and piezoelectric sensor are shown in Figure 10. The output of piezoelectric sensor is sampled by the oscilloscope at a sample rate of 1000 Hz. The output of quartz flexure accelerometer is sampled at a synchronized rate of 1000 Hz, and then down sampled to 100 Hz. Hence, the ratio of the high sample rate to the low sample rate is 10. These two sensors are tested by a sinusoid acceleration signal generated by the shaking table. The frequency of the sinusoid signal is 40 Hz, and the peak-to-peak amplitude is 2 g. Limited by the memory depth, the total sampling time is 2.2 s. Still, the drifts of these two sensors can be compared. By applying a linear fitting to the data of piezoelectric sensor, the drift of piezoelectric sensor is 2.2 g/s. The output of quartz flexure accelerometer keeps stable in this very short time.

**Figure 11:**
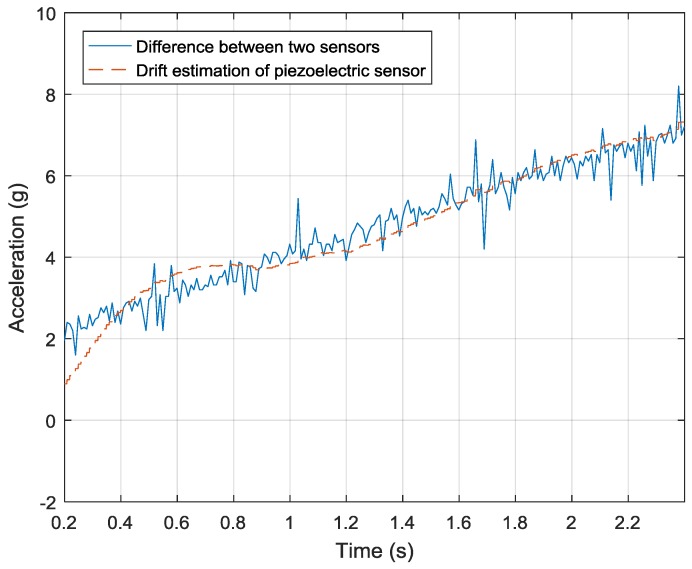
Drift estimation of piezoelectric sensor.

The result of the drift-state estimation process is shown in Figure 11. Subtracting the data of quartz flexure accelerometer from the data of piezoelectric sensor gives the observation data, which is the blue solid line shown in Figure 11. The first-stage Kalman filter investigated in simulation is applied to this observation data. Several key parameters of this Kalman filter are modified according to the experimental data. The observation covariance matrix is changed to 0.11 as the RMSE of observation data is 0.33 g. The covariance matrix of state transmission error is assigned 1 × 10^−6^ along the diagonals and 0 elsewhere, because the state vector keeps stable in this very short time. The state vector starts from a random vector initially, and it ends to be ${\left[0.27-1.09\;0.41\;4.53\right]}^{\prime }$ at time 2.4 s. The drift estimation of piezoelectric sensor, which is plotted by red dashed line, converges to the trend of drift as more observation data are obtained.

**Figure 12:**
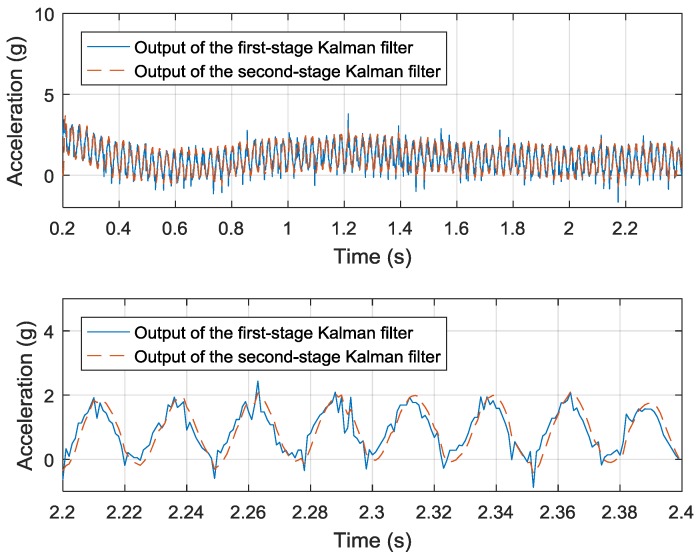
The results of two Kalman filters on a time scale of 2.2 s and 0.2 s.

After the drift of piezoelectric sensor is removed, a linear-prediction-based Kalman filter is applied to reduce the measurement noise, which is shown in Figure 12. The order of the linear prediction model is 2. The standard deviation of measurement error is set to be 1e-1 according to the experimental data. The standard deviation of prediction error is set to be 1 × 10^−2^ rather than 1 × 10^−4^ that derived from simulation, since the sample rate is 1 kHz in the experiment rather than 10 kHz that used in the simulation. Compared with the drift-removed data given by the first-stage Kalman filter, the output of linear-prediction has a smaller measurement noise. Since the real acceleration of the shaking table is unavailable, the improvement of measurement noise is not calculated. By using the cross correlation method, the latency between the drift-removed data and the output of the second-stage Kalman filter is 1 sample.

In summary, the dual-sensor vibration measuring system achieves the low-drift and high-dynamic performance that required by early failure detection for mining machines. The simulation results show that the drift of high-dynamic sensor can be estimated and removed by fusing measurements from the low-drift sensor and the high-dynamic sensor. In the low frequency band below 1 Hz, more than 34 dB improvement is achieved for the signals that vary from 10 Hz to 100 Hz. In the high frequency band, measurement noise has improved more than 9.3 dB through using linear-prediction-based Kalman filter. The validity of simulation results is verified by experiments. A quartz flexure accelerometer is used as the low-drift sensor, and a piezoelectric sensor is used as the high-dynamic sensor. The experimental results show that the drift of high-dynamic sensor, which reaches 2.2 g/s, is much bigger than low-drift sensor. After the drift estimation process, the drift of high-dynamic sensor converges to the drift of low-drift sensor, which keeps stable during a long time. Hence, the lower frequency boundary of high-dynamic sensor is extended. Also, the measurement noise is reduced as demonstrated by simulation. The parameters used in experiments are reasonable.

## 6. Conclusions

This paper presented a dual-sensor-based hardware structure and processing algorithms. The hardware structure comprises two sensors (a high-dynamic sensor and a low-drift sensor) that work at different sample rates but are synchronized through using the same driving clock. The processing algorithms, including drift-state estimation and a linear-prediction-based Kalman filter, were verified through simulation and experiment. The simulation and experimental results showed that combined high-dynamic and low-drift measuring ability was achieved. Future work involves applying this method to an online condition monitoring system.

## Figures and Tables

**Table 1:** Anti-drift improvements for different signal bands.

Band (Hz)	Improvement (dB)
10	34
50	34
100	40
